# Supplementary material for: Relationship between health literacy and attitudes toward acupuncture: A web-based cross-sectional survey with a panel of Japanese residents
Source: PLoS One. 2023 Oct 20;18(10):e0292729. doi: 10.1371/journal.pone.0292729 (PMC10588898; doi:10.1371/journal.pone.0292729)
Supplement: S1 Table — (DOCX) [file pone.0292729.s001.docx]

S1 Table. STROBE Statement—checklist of items that should be included in reports of observational studies

|  | Item No. | Recommendation | Page  No. | Relevant text from manuscript |
| --- | --- | --- | --- | --- |
| **Title and abstract** | 1 | (*a*) Indicate the study’s design with a commonly used term in the title or the abstract | 1 | Relationship between health literacy and attitudes toward acupuncture: A web-based cross-sectional survey with a panel of Japanese residents |
|  |  | (*b*) Provide in the abstract an informative and balanced summary of what was done and what was found | 2,3 | The relationship between health literacy of Japanese people, their attitudes toward acupuncture, and their behavior in choosing this therapy is currently unclear. Therefore, for this study, we conducted a web-based survey to address this unknown relationship. A questionnaire comprising four categories (health status, health literacy, previous acupuncture experience, and attitudes toward acupuncture) was administered to 1,600 Japanese participants. For this study, we performed cross-tabulation and path analysis to examine the relationship between each questionnaire item. The mean score of participants’ health literacy was 3.41 (SD = 0.74), and older, educated, female participants tended to have higher health literacy. The respondents perceived acupuncture to be effective for chronic low back pain, tension-type headache, and knee pain due to osteoarthritis (40.0%, 38.7%, and 21.8%, respectively). Contrastingly, acupuncture was perceived as far less effective for postoperative nausea/vomiting and prostatitis symptoms (8.4% and 8.7%, respectively). Of the total study respondents, 34.4% reported that they would try acupuncture only if recommended by clinical practice guidelines, and 35.6% agreed that acupuncture is safe. The path analysis showed that attitudes toward acupuncture were significantly influenced by the participants’ health literacy, number of information sources, and previous acupuncture experience. However, it was also found that experience with acupuncture was not directly associated with health literacy.  Although the Japanese population with higher health literacy is more likely to perceive acupuncture positively, they do not necessarily have sufficient relevant knowledge of the clinical evidence. Therefore, their decision to receive acupuncture may be more dependent on personal narratives rather than clinical evidence. Thus, future challenges lie in individual education of the population on how to choose a reliable health information source, and organizational efforts to provide more reliable health information. |
| **Introduction** |  |  |  |  |
| Background/rationale | 2 | Explain the scientific background and rationale for the investigation being reported | 4–6 | Previous studies suggest that low health literacy is associated with poorer health outcomes and higher costs, such as more hospitalizations, greater use of emergency services and higher mortality rates in older people [3–5]. Studies including measurement of health literacy have been conducted, particularly in the United States, although the evidence on the relationship between health literacy and health disparities is still scarce and limited [6].  In Japan, health literacy scales with good reliability and validity have already been developed or translated by previous studies, and Japanese people’s health literacy has been assessed using those scales [7–9]. It has been reported that Japanese people with higher health literacy are more likely to obtain sufficient health information from multiple sources and, thus, are less likely to engage in risky habits like smoking and regular drinking [10]. However, a survey has found that Japanese health literacy is relatively lower than that of Europeans [9]. The authors who conducted that survey speculate that this may be due in part to an insufficient primary health care system and difficulty accessing reliable health information, but no solid factors have been identified. Moreover, it is unknown whether the health literacy of the Japanese people influences the use of healthcare measures for which the evidence is controversial and not well established. Representative of such healthcare measures would be the area of healthcare grouped as complementary therapies [11,12]. Studies in the United States suggest that people with high health literacy tend to use complementary therapies at a higher rate [13,14]. In contrast, to our knowledge, no such studies have been reported from East Asian countries including Japan, China and South Korea.  One typical example of complementary therapies with insufficiently established evidence that has been used by some percentage of the Japanese population is acupuncture therapy. It has been reported that in Japan, approximately 5–7% of people receive acupuncture at least once a year [15,16]. Musculoskeletal problems were the most common condition for which acupuncture was used (approximately 80%), and referral by family or friends was the most common reasons for people receiving acupuncture (approximately 60%) [16]. Around the 2000s, it was unknown to what extent information on the evidence for acupuncture was widespread within the Japanese population; additionally, it was unclear how much their health literacy played a role in their decision to use acupuncture for their intended condition. Incidentally, existing survey reports suggest that Japanese users of acupuncture tend to have lower education levels [15,16], but these results were probably confounded by age factor because the older population, who were relatively less educated, were more likely to use acupuncture than the younger [16]. Moreover, the relationship between health literacy and education levels has been controversial among scholars in Japan [9,10,17]. |
| Objectives | 3 | State specific objectives, including any prespecified hypotheses | 6–7 | Recently, studies have found some evidence of the clinical effectiveness and safety of acupuncture. Albeit insufficient, there are some positive conclusions regarding acupuncture for several health conditions like chronic low back pain and headaches in the Cochrane Database of Systematic Reviews [18–23]. Furthermore, several evidence-based clinical practice guidelines have been developed in Japan that include recommendations for acupuncture [24]. Under these circumstances, it remains unknown whether people with high health literacy adequately scrutinize the evidence, affirm acupuncture for the conditions for which evidence is shown, and consequently receive this treatment.  In this study, we conducted a web-based questionnaire survey in Japan to address the gap in this body of research. The aim of the study was to investigate the relationship between people’s health literacy and their health information sources and their attitudes toward acupuncture. In addition, this study aimed to understand the interrelationships among multiple factors such as sociodemographic characteristics, health literacy, health status, information sources, attitudes toward acupuncture and experience of acupuncture. Finally, to the WHO policy which encourages member states to provide individuals with reliable information on the benefits and risks of integrating traditional and complementary medicine into their healthcare system [25], our study can contribute insights for further understanding of and research on relevant education measures for the public in terms of health literacy. |
| **Methods** |  |  |  |  |
| Study design | 4 | Present key elements of study design early in the paper | 7 | For this study, we conducted a web-based cross-sectional questionnaire survey between January 27 and February 3, 2020. |
| Setting | 5 | Describe the setting, locations, and relevant dates, including periods of recruitment, exposure, follow-up, and data collection | 7–8 | For this study, we conducted a web-based cross-sectional questionnaire survey between January 27 and February 3, 2020. The survey was commissioned to Mellinks Co., Ltd., Tokyo, Japan (https://www.mellinks.co.jp/), an internet research company that has nationwide panels by age group.  This was a closed, panel-based survey that required registration for eligible respondents to be able to complete it. It was conducted on the Mellinks website for monitors registered with Mellinks’ affiliated research firm. |
| Participants | 6 | (*a*) *Cross-sectional study*—Give the eligibility criteria, and the sources and methods of selection of participants | 7 | (Same as above) |
|  |  | (*b*) *Cohort study*—For matched studies, give matching criteria and number of exposed and unexposed  *Case-control study*—For matched studies, give matching criteria and the number of controls per case | n/a | n/a |
| Variables | 7 | Clearly define all outcomes, exposures, predictors, potential confounders, and effect modifiers. Give diagnostic criteria, if applicable | 9,10 | We developed a questionnaire comprising four categories (S4 Table): health status (Q1, 2), health literacy (Q3, 4), experience of receiving acupuncture (Q5, 6), and recognition and choice behavior regarding acupuncture (Q7–11).  Along with the abovementioned 11 questions, we created questions for basic information on sociodemographic attributes like sex, age, educational attainment, occupation, and residential area. |
| Data sources/ measurement | 8 | For each variable of interest, give sources of data and details of methods of assessment (measurement). Describe comparability of assessment methods if there is more than one group | 7,8 | This was a closed, panel-based survey that required registration for eligible respondents to be able to complete it. It was conducted on the Mellinks website for monitors registered with Mellinks’ affiliated research firm  Those who agreed to participate in this study were asked to provide their educational attainment and occupation, which indicated the completion of the preliminary survey. Thereafter, Mellinks notified the consenting participants via e-mail or web-based invitation, before commencing the survey. All 11 questions were displayed on a single web page, and the participants were asked to answer all. |
| Bias | 9 | Describe any efforts to address potential sources of bias | 8–11 | To prevent missing data, this question session was designed in a manner that it will be considered as incomplete if there were unanswered questions. Once the participants had answered all the questions, they were asked to review their answers again and were allowed to revise them, if required.    The developed questionnaire draft was pre-tested using our university staff members who were not included in the study panel to make it easier to answer. Based on the inconvenience pointed out by several of the staff members, we improved the questionnaire to arrive at the final version. Thereafter, the completed questionnaire was arranged by Mellinks for the web survey. We checked the usability of the survey screen before releasing it to the participants. |
| Study size | 10 | Explain how the study size was arrived at | 11 | The lifetime use of acupuncture in Japan has been reported to be approximately 25% [16]. Assuming a confidence level of 95%, an acceptable sampling error of 5%, and a response rate of 50%, 384 samples were required for that 25% in this study (n = 1.962 x 0.5(1-0.5)/0.052). Therefore, we decided to collect 1,600 samples to ensure reliability. |
| Quantitative variables | 11 | Explain how quantitative variables were handled in the analyses. If applicable, describe which groupings were chosen and why | 11,12 | Based on responses to Q3 (health literacy measures), respondents were divided into two groups of health literacy according to the median score of five items (5-point rating each): a median score of four or more was regarded as the higher health literacy (HHL) group, and that of less than four, as the lower health literacy (LHL) group [7]. For Q8–10 which assessed the participants’ attitude toward acupuncture, respondents were divided into two groups of affirmation on acupuncture based on each respondent’s 5-point ratings; a score of four or more was regarded as the acupuncture approval (AA) group and that of less than four as the acupuncture disapproval/Neutral (AD/N) group. In Q8, the division of the respondents into two groups (AA and AD/N group) depended on whether their median score for six symptoms was four or higher.  Using these classifications, we assessed the interrelationships among sex, health literacy, experience of acupuncture, and attitudes toward acupuncture through cross-tabulation. Pearson’s chi-squared test was used for these analyses. Odds ratios and 95% confidence intervals were shown as effect sizes. |
| Statistical methods | 12 | (*a*) Describe all statistical methods, including those used to control for confounding | 11–13 | Additionally, we performed a path analysis to examine direct and indirect interrelationships among sociodemographic factors (sex, age, and educational attainment), health literacy, the number of information sources, health status, and experience and attitudes toward acupuncture among the participants. For health literacy, we used continuous variables, while dichotomized data were used for cross-tabulation. Model parameters were estimated using the maximum-likelihood estimation. The estimation was performed by assuming endogenous correlations. For this, first we performed a path analysis to test the hypothesis model established based on the results of above cross-tabulation and previous studies suggesting a relationship between health literacy and several factors, like sociodemographic characteristics or the number of information sources [10], then trimmed non-significant paths to reach a final model. The fitness of the model was evaluated using comparative fit index (CFI) and root mean square error of approximation (RMSEA). If the CFI was larger than 0.95 and the RMSEA was < 0.05, the model was considered to be acceptable [32].  All statistical analyses were performed using the software, Jamovi Version 2.3.0 [33]. The analysis was conducted without weighting the sample. For path analysis, we used Jamovi’s modules of PATH ANALYSIS 0.8.0. The significance level was set at p < 0.05. |
|  |  | (*b*) Describe any methods used to examine subgroups and interactions | n/a | n/a |
|  |  | (*c*) Explain how missing data were addressed | n/a | n/a |
|  |  | (*d*) *Cohort study*—If applicable, explain how loss to follow-up was addressed  *Case-control study*—If applicable, explain how matching of cases and controls was addressed  *Cross-sectional study*—If applicable, describe analytical methods taking account of sampling strategy | n/a | n/a |
|  |  | (*e*) Describe any sensitivity analyses | n/a | n/a |
| **Results** |  |  |  |  |
| Participants | 13 | (a) Report numbers of individuals at each stage of study—eg numbers potentially eligible, examined for eligibility, confirmed eligible, included in the study, completing follow-up, and analysed | 8,14 | Prior to the questionnaire survey, approximately 40,000 potential survey monitors were randomly selected and contacted via e-mail or web notification with the survey protocol. The protocol notice provided an overview of the study design, including the name of the study investigator, on a webpage, and confirmed the participants’ willingness to participate in the study.  The total number of those who agreed to participate at the pre-survey stage was 3,292. Among them, the collection of responses continued until the number of respondents reached 160 in each of the age and sex quotas, and a total of 1,600 responses were finally collected. Therefore, the completion rate was 48.6%. |
|  |  | (b) Give reasons for non-participation at each stage | n/a | n/a |
|  |  | (c) Consider use of a flow diagram | n/a | n/a |
| Descriptive data | 14 | (a) Give characteristics of study participants (eg demographic, clinical, social) and information on exposures and potential confounders | 15–17 | (Shown in Table 1: Sociodemographic characteristics of the respondents.) |
|  |  | (b) Indicate number of participants with missing data for each variable of interest | n/a | n/a |
|  |  | (c) *Cohort study*—Summarise follow-up time (eg, average and total amount) | n/a | n/a |
| Outcome data | 15 | *Cohort study*—Report numbers of outcome events or summary measures over time | n/a | n/a |
|  |  | *Case-control study—*Report numbers in each exposure category, or summary measures of exposure | n/a | n/a |
|  |  | *Cross-sectional study—*Report numbers of outcome events or summary measures | n/a | n/a |
| Main results | 16 | (*a*) Give unadjusted estimates and, if applicable, confounder-adjusted estimates and their precision (eg, 95% confidence interval). Make clear which confounders were adjusted for and why they were included | 13 | The analysis was conducted without weighting the sample. |
|  |  | (*b*) Report category boundaries when continuous variables were categorized | 11,12 | Based on responses to Q3 (health literacy measures), respondents were divided into two groups of health literacy according to the median score of five items (5-point rating each): a median score of four or more was regarded as the higher health literacy (HHL) group, and that of less than four, as the lower health literacy (LHL) group [7]. For Q8–10 which assessed the participants’ attitude toward acupuncture, respondents were divided into two groups of affirmation on acupuncture based on each respondent’s 5-point ratings; a score of four or more was regarded as the acupuncture approval (AA) group and that of less than four as the acupuncture disapproval/Neutral (AD/N) group. In Q8, the division of the respondents into two groups (AA and AD/N group) depended on whether their median score for six symptoms was four or higher. |
|  |  | (*c*) If relevant, consider translating estimates of relative risk into absolute risk for a meaningful time period | n/a | n/a |
| Other analyses | 17 | Report other analyses done—eg analyses of subgroups and interactions, and sensitivity analyses | n/a | n/a |
| Key results | 18 | Summarise key results with reference to study objectives | 35 | To the best of our knowledge, this is the first study to simultaneously question the Japanese population on health literacy items and their experience of and attitudes toward acupuncture. The study found that overall, the health literacy of Japanese respondents tended to increase with age, and the main sources of health information differed by generation with respect to SNS, medical doctors, and newspapers. The annual use of acupuncture was 8.2% (95% CI: 6.9–9.6), which was slightly higher than that reported in previous studies, being 6.7% (95% CI: 5.2–8.2) in 2000 [15] and 6.7% (95% CI: 5.2–8.3) in 2005 [16]. Since our survey panel is not proportional to the population of Japan, it is difficult to directly compare previous studies with that of ours. Nevertheless, it is obvious that the annual use of acupuncture in Japan is higher than that in almost all Western countries [35,36]. |
| Limitations | 19 | Discuss limitations of the study, taking into account sources of potential bias or imprecision. Discuss both direction and magnitude of any potential bias | 40,41 | This study had some limitations. First, because the number of respondents in each age and sex group was 160, it was not proportional to the population structure of Japan. Second, the respondents were not selected through random sampling, which is a common issue in web-based surveys using panel respondents. Third, it is difficult to know the classification and reasons of those who agreed to respond but were not included among the respondents (1,692 in this study), a limitation that is inherent to panel-based studies. Fourth, we did not collect data on the respondents’ income, which may have influenced the path analysis, particularly in terms of education, health literacy, and experience of acupuncture. Fifth, we did not focus on the relationship and influence of the type of comorbidities because the respondents were not proportional to the Japanese population, as mentioned above, and morbidity was less prevalent than we expected. Sixth, for the 25.4% of respondents who had received acupuncture in their lifetime, their attitudes toward this therapy might have been influenced by actual positive or negative experiences, rather than their health literacy. However, detailed questions on how those who received acupuncture in the past felt about the treatment were not included in this survey. Seventh, the measurement of health literacy was based on self-reporting, which may introduce biases, such as recall bias and over- or underestimation.  Despite these limitations, this is the first study to assess the relationship between health literacy and attitudes toward acupuncture, and it provides useful suggestions for the future of health education. |
| Interpretation | 20 | Give a cautious overall interpretation of results considering objectives, limitations, multiplicity of analyses, results from similar studies, and other relevant evidence | 41,42 | In this study, involving a panel of 1,600 respondents stratified by age and sex, we found that age, educational attainment, and sex might be associated with the health literacy of Japanese people. Older, educated, female participants tended to have higher health literacy. Furthermore, it can be deduced that consulting medical doctors and reading newspapers may have influenced the level of participants’ health literacy. Although Japanese people with higher health literacy are more likely to perceive acupuncture positively, they do not necessarily have sufficient knowledge of the clinical evidence of acupuncture, and their decision to receive acupuncture may be more dependent on personal narratives from their family and acquaintances rather than clinical research evidence. |
| Generalisability | 21 | Discuss the generalisability (external validity) of the study results | 35 | Since our survey panel is not proportional to the population of Japan, it is difficult to directly compare previous studies with that of ours. Nevertheless, it is obvious that the annual use of acupuncture in Japan is higher than that in almost all Western countries [35,36]. |
| **Other information** |  |  |  |  |
| Funding | 22 | Give the source of funding and the role of the funders for the present study and, if applicable, for the original study on which the present article is based |  | This research was funded by the 2018 President’s Incentive Research Grant from Morinomiya University of Medical Sciences (YO). The funder had no role in the study design, data collection, analysis, decision to publish, or manuscript preparation. |
