# Supplementary material for: Relationship between health literacy and attitudes toward acupuncture: A web-based cross-sectional survey with a panel of Japanese residents
Source: PLoS One. 2023 Oct 20;18(10):e0292729. doi: 10.1371/journal.pone.0292729 (PMC10588898; doi:10.1371/journal.pone.0292729)
Supplement: S2 Table — (DOCX) [file pone.0292729.s002.docx]

**S2 Table. Checklist for Reporting Results of Internet E-Surveys (CHERRIES)**

| **Item Category** | **Checklist Item** | **Explanation** |
| --- | --- | --- |
| Design | Describe survey design | **“Study populations and procedures” in Materials and methods**  This was a closed, panel-based survey that required registration for eligible respondents to be able to complete it. It was conducted on the Mellinks website for monitors registered with Mellinks’ affiliated research firm. |
| IRB (Institutional Review  Board) approval and informed  consent process | IRB approval | **“Ethical statement” in Materials and methods**  The study protocol was approved by the Ethics Committee of Morinomiya University of Medical Sciences (No. 2019-100). |
|  | Informed consent | **“Ethical statement” in Materials and methods**  The research panel members were informed of the objective, content, approximate time required for response, and protection policy of personal information, and the fact that participation was not forced prior to starting the questionnaire. When the panel members agreed to become respondents with a written document on the web page, they clicked on an agreement button, which started the questionnaire survey. |
|  | Data protection | **“Ethical statement” in Materials and methods**  The authors had no special access privileges to the data that could identify individual respondents. |
| Development and pre-testing | Development and testing | **“Development of questionnaire”** **in Materials and methods**  We developed a questionnaire comprising four categories (S4 Table): health status (Q1, 2), health literacy (Q3, 4), experience of receiving acupuncture (Q5, 6), and recognition and choice behavior regarding acupuncture (Q7–11). Regarding the health literacy measurement of Q3, we used a 5-item questionnaire developed by Ishikawa et al., who are considered to be specialists in public health who conducted a pilot study questioning 190 male office workers during an annual health check-up. The scale items of the questionnaire draft were constructed to directly reflect the WHO definitions [7]. Each item was rated on a 5-point scale, ranging from 1 (strongly disagree) to 5 (strongly agree). We chose this scale [7] for this study because of the following reasons: the internal reliability of the scale has been evaluated and warranted (Cronbach’s α = 0.86); it comprises only five questions, making it compact and user-friendly for respondents; it was subsequently used in some surveys targeting a bigger sample of the Japanese population [29,30].  As for the recognition and choice behavior of acupuncture, the questionnaire asked for a reliable information source (Q7), expected health conditions that may benefit from acupuncture (Q8), possible influence of clinical practice guidelines on the decision to receive acupuncture (Q9), and the safety of acupuncture (Q10,11). For Q8–10, we used a 5-point rating scale similar to Q3. For Q8 (“Do you think that acupuncture is effective for the following symptoms or diseases?”), we selected six conditions for which the Cochrane Database of Systematic Reviews concluded positively on the clinical benefit of acupuncture as of January 2020 [19–23,31].  Along with the abovementioned 11 questions, we created questions for basic information on sociodemographic attributes like sex, age, educational attainment, occupation, and residential area. The developed questionnaire draft was pre-tested using our university staff members who were not included in the study panel to make it easier to answer. Based on the inconvenience pointed out by several of the staff members, we improved the questionnaire to arrive at the final version. Thereafter, the completed questionnaire was arranged by Mellinks for the web survey. We checked the usability of the survey screen before releasing it to the participants. |
| Recruitment process and description  of the sample having access  to the questionnaire | Open survey versus closed survey | **“Study populations and procedures” in Materials and methods**  The survey was commissioned to Mellinks Co., Ltd., Tokyo, Japan (https://www.mellinks.co.jp/), an internet research company that has nationwide panels by age group.  This was a closed, panel-based survey that required registration for eligible respondents to be able to complete it. It was conducted on the Mellinks website for monitors registered with Mellinks’ affiliated research firm. |
|  | Contact mode | **“Study populations and procedures” in Materials and methods**  Prior to the questionnaire survey, approximately 40,000 potential survey monitors were randomly selected and contacted via e-mail or web notification with the survey protocol. The protocol notice provided an overview of the study design, including the name of the study investigator, on a webpage, and confirmed the participants’ willingness to participate in the study. |
|  | Advertising the survey | (Same as above) |
| Survey administration | Web/E-mail | **“Study populations and procedures” in Materials and methods**  Prior to the questionnaire survey, approximately 40,000 potential survey monitors were randomly selected and contacted via e-mail or web notification with the survey protocol. The protocol notice provided an overview of the study design, including the name of the study investigator, on a webpage, and confirmed the participants’ willingness to participate in the study.  Thereafter, Mellinks notified the consenting participants via e-mail or web-based invitation, before commencing the survey. All 11 questions were displayed on a single web page, and the participants were asked to answer all. |
|  | Context | **“Study populations and procedures” in Materials and methods**  This was a closed, panel-based survey that required registration for eligible respondents to be able to complete it. It was conducted on the Mellinks website for monitors registered with Mellinks’ affiliated research firm. |
|  | Mandatory/voluntary | **“Study populations and procedures” in Materials and methods**  Those who completed this survey were compensated; however, the exact amount of compensation was not disclosed by the research firm that was affiliated with Mellinks. |
|  | Incentives | **“Study populations and procedures” in Materials and methods**  Incidentally, the typical compensation for such surveys ranges from a few yen to 10 yen for the preliminary survey and from 50 yen to 100 yen for the actual survey. |
|  | Time/Date | **“Study populations and procedures” in Materials and methods**  For this study, we conducted a web-based cross-sectional questionnaire survey between January 27 and February 3, 2020. |
|  | Randomization of items or questionnaires | Not applicable |
|  | Adaptive questioning | Details of the questions are shown in the S4 table. |
|  | Number of Items | **“Study populations and procedures” in “Study populations and procedures” in Materials and methods**  All 11 questions were displayed on a single web page, and the participants were asked to answer all. |
|  | Number of screens (pages) | (Same as above) |
|  | Completeness check | **“Study populations and procedures” in Materials and methods**  To prevent missing data, this question session was designed in a manner that it will be considered as incomplete if there were unanswered questions. |
|  | Review step | **“Study populations and procedures” in Materials and methods**  Once the participants had answered all the questions, they were asked to review their answers again and were allowed to revise them, if required. |
| Response rates | Unique site visitor | Not applicable |
|  | View rate (Ratio of unique survey visitors/unique site visitors) | Not applicable |
|  | Participation rate (Ratio of unique visitors who agreed to participate/unique first survey  page visitors) | Not applicable |
|  | Completion rate (Ratio of users who finished the survey/users who agreed to participate) | **Results**  The total number of those who agreed to participate at the pre-survey stage was 3,292. Among them, the collection of responses continued until the number of respondents reached 160 in each of the age and sex quotas, and a total of 1,600 responses were finally collected. Therefore, the completion rate was 48.6%. |
| Preventing multiple entries from  the same individual | Cookies used | Not applicable |
|  | IP check | Not applicable |
|  | Log file analysis | Not applicable |
|  | Registration | **“Study populations and procedures” in Materials and methods**  This was a closed, panel-based survey that required registration for eligible respondents to be able to complete it. It was conducted on the Mellinks website for monitors registered with Mellinks’ affiliated research firm. |
| Analysis | Handling of incomplete questionnaires | **“Study populations and procedures” in Materials and methods**  To prevent missing data, this question session was designed in a manner that it will be considered as incomplete if there were unanswered questions. |
|  | Questionnaires submitted with an atypical timestamp | Not applicable |
|  | Statistical correction | **“Data analysis” in Materials and methods**  The analysis was conducted without weighting the sample. |
