# Supplementary material for: Relationship between health literacy and attitudes toward acupuncture: A web-based cross-sectional survey with a panel of Japanese residents
Source: PLoS One. 2023 Oct 20;18(10):e0292729. doi: 10.1371/journal.pone.0292729 (PMC10588898; doi:10.1371/journal.pone.0292729)
Supplement: S3 Table — (DOCX) [file pone.0292729.s003.docx]

**S3 Table. Checklist of reporting guidelines for structural equation modelling**

| **Item No.** | **Recommendation** | **Relevant text from manuscript** |
| --- | --- | --- |
| 1 | As determined by an a priori power analysis, the minimum number of participants needed, given the models that are being tested. | Not applicable |
| 2 | At least one alternative model that is plausible in light of extant theory or relevant empirical findings. | **“Data analysis” in Materials and methods**  For this, first we performed a path analysis to test the hypothesis model established based on the results of above cross-tabulation and previous studies suggesting a relationship between health literacy and several factors, like sociodemographic characteristics or the number of information sources [10], then trimmed non-significant paths to reach a final model. |
| 3 | Graphical displays of all measurement and structural models. | They are shown in Fig 4 and S5 Fig. |
| 4 | Brief details about the psychometric properties of scale scores for all measured variables (e.g., Cronbach’s alpha and its 95% confidence intervals or, preferably, omega as well as 2 to 3 sentences per measure detailing evidence of content and construct validities). | **“Development of questionnaire” in Materials and methods**  We chose this scale [7] for this study because of the following reasons: the internal reliability of the scale has been evaluated and warranted (Cronbach’s α = 0.86); it comprises only five questions, making it compact and user-friendly for respondents; it was subsequently used in some surveys targeting a bigger sample of the Japanese population [29,30]. |
| 5 | The proportion of data that are missing and whether missing data are MCAR, MAR, or MNAR. As well, researchers should explicate how this decision was reached (e.g., why does a researcher assume missing data are MAR?), and the action taken to address missing data. | **“Study populations and procedures” in Materials and methods**  To prevent missing data, this question session was designed in a manner that it will be considered as incomplete if there were unanswered questions. Once the participants had answered all the questions, they were asked to review their answers again and were allowed to revise them, if required. The survey targeted 160 men and 160 women in each age group (20s (20–29 years), 30s (30–39 years), 40s (40–49 years), 50s (50–59 years) and 60s (60–69 years)).  Thus, a total of 1,600 participants living in Japan were included in this study. When 160 respondents in each age and sex group responded, the respective quota was closed. Responses were tabulated automatically. |
| 6 | Assessments of univariate and multivariate normality for all measured indicators. | Not applicable |
| 7 | The estimation method used to generate all SEMs (default is ML estimation). | **“Data analysis” in Materials and methods**  Model parameters were estimated using the maximum-likelihood estimation. The estimation was performed by assuming endogenous correlations. |
| 8 | The software (including version) that was used to analyze the data. | **“Data analysis” in Materials and methods**  For path analysis, we used Jamovi’s modules of PATH ANALYSIS 0.8.0. |
| 9 | In accordance with the advised two-step approach, full CFA details about each measurement model followed by complete SEM details about the structural model. | Not applicable |
| 10 | Indicator and composite reliabilities. | Not applicable |
| 11 | Average variance extracted (AVE) for each latent factor which denote convergent validity. | Not applicable |
| 12 | Discriminant validity of latent factors, as per Fornell and Larcker’s (1981) test. | Not applicable |
| 13 | All standardized loadings from latent variables to manifest variables (reflective models). | Not applicable |
| 14 | Fit indices that reflect overall, absolute, and incremental fit. If applicable, predictive fit indicators should be included. | **“Path analysis” in Results**  Confirming that the fitness indices of the hypothesis model (S5 Fig) were adequate (CFI = 0.968 and RMSEA = 0.038 [95% CI: 0.030–0.048]), the final model was developed after removing the non-significant path, as shown in Fig 4. It was noted that the model fitness indices improved [CFI = 0.978 and RMSEA = 0.031 (95% CI: 0.022–0.040)]. |
| 15 | A clear and compelling rationale for all post-hoc model modifications. | **“Data analysis” in Materials and methods**  For this, first we performed a path analysis to test the hypothesis model established based on the results of above cross-tabulation and previous studies suggesting a relationship between health literacy and several factors, like sociodemographic characteristics or the number of information sources [10], then trimmed non-significant paths to reach a final model. |
| 16 | An indicator of effect size for the final model. | **Fig 4. Path analysis (final model).**  The numbers shown correspond to a standardized path coefficient. |
