# Supplementary material for: Relationship between health literacy and attitudes toward acupuncture: A web-based cross-sectional survey with a panel of Japanese residents
Source: PLoS One. 2023 Oct 20;18(10):e0292729. doi: 10.1371/journal.pone.0292729 (PMC10588898; doi:10.1371/journal.pone.0292729)
Supplement: S4 Table — This questionnaire comprises four categories of health status (Q1, 2), health literacy (Q3, 4), experience of receiving acupuncture (Q5, 6) and recognition and choice behavior about acupuncture (Q7–11). Regarding the health literacy measurement of Q3, we used a 5-item questionnaire developed by Ishikawa et al. [7]. (DOCX) [file pone.0292729.s004.docx]

**S4 Table. 11-item questionnaire**

| Question number | Question | Answer | | | | |
| --- | --- | --- | --- | --- | --- | --- |
| １ | Do you think you are healthy? | Strongly disagree | Disagree | Neither | Agree | Strongly Agree |
| 2 | Are there any conditions or diseases that you are currently treating? | Free description | | | | |
| 3 | If you need, do you think you would be able to find and use information related to illness and health on your own? |  |  |  |  |  |
|  | (i) collect health-related information from various sources, | Strongly disagree | Disagree | Neither | Agree | Strongly Agree |
|  | (ii) extract the information s/he wanted, |  |  |  |  |  |
|  | (iii) understand and communicate the obtained information, |  |  |  |  |  |
|  | (iv) consider the credibility of the information and |  |  |  |  |  |
|  | (v) make decisions based on the information, specifically in the context of health-related issues. |  |  |  |  |  |
| 4 | How do you get information related to illness and health? Please choose any number from the following. | Television  Radio  Internets and Blogs (except for social networking sites, SNS)  SNS (Twitter, Facebook, Instagram)  Newspapers  Magazines  Books  Family, Friends, and Acquaintances  Medical doctors  Medical professionals other than medical doctors  Others | | | | |
| 5 | Have you ever received acupuncture and moxibustion treatment? | Currently receiving  Not currently receiving, but have received within the past year  Have not received within the past year, but have received in the past  Have never received | | | | |
| 6 | If you answered "have never received" to Q5, please choose the reason from the following (multiple answers are possible) | Because I was healthy  Because I did not feel the necessity  Because I did not expected efficacy of acupuncture  Because acupuncture seemed painful  Because I thought acupuncture is a dubious treatment  Because I was afraid of the side effects  Because I thought it would be expensive  Because I did not know about acupuncture  Others | | | | |
| 7 | Which source of information do you place confidence when deciding whether or not to have acupuncture treatment? (multiple answers allowed) | Television  Radio  Internets and Blogs (except for social networking service, SNS)  SNS (Twitter, Facebook, Instagram, etc)  Newspapers  Magazines  Books  Family, Friends, and Acquaintances  Medical doctors  Medical professionals other than medical doctors  Clinical Practice Guidelines  Not sure  Others | | | | |
| 8 | Do you think that acupuncture is effective for the following symptoms or diseases? |  |  |  |  |  |
|  | ・Chronic low back pain | Strongly disagree | Disagree | Neither/not sure | Agree | Strongly Agree |
|  | ・Migraine (prevention and relief of attacks) |  |  |  |  |  |
|  | ・Tension-type headache (associated with neck and shoulder stiffness) |  |  |  |  |  |
|  | ・Postoperative nausea and vomiting |  |  |  |  |  |
|  | ・Prostatitis symptoms |  |  |  |  |  |
|  | ・Knee pain due to osteoarthritis |  |  |  |  |  |
| 9 | In recent years, Clinical Practice Guidelines which recommend medical options based on latest scientific evidence to make appropriate choices between medical professionals and patients have become popular. For example, if acupuncture is recommended in a Clinical Practice Guidelines about your conditions or diseases, are you going to try acupuncture? | Strongly disagree | Disagree | Neither | Agree | Strongly Agree |
| 10 | Do you think that acupuncture is safe? | Strongly disagree | Disagree | Neither | Agree | Strongly Agree |
| 11 | If you answered “Strongly disagree” or “Disagree” to Q10, what do you consider unsafe? | Free description | | | | |
