# Supplementary figures and images for: Relationship between health literacy and attitudes toward acupuncture: A web-based cross-sectional survey with a panel of Japanese residents
Source: PLoS One. 2023 Oct 20;18(10):e0292729. doi: 10.1371/journal.pone.0292729 (PMC10588898; doi:10.1371/journal.pone.0292729)

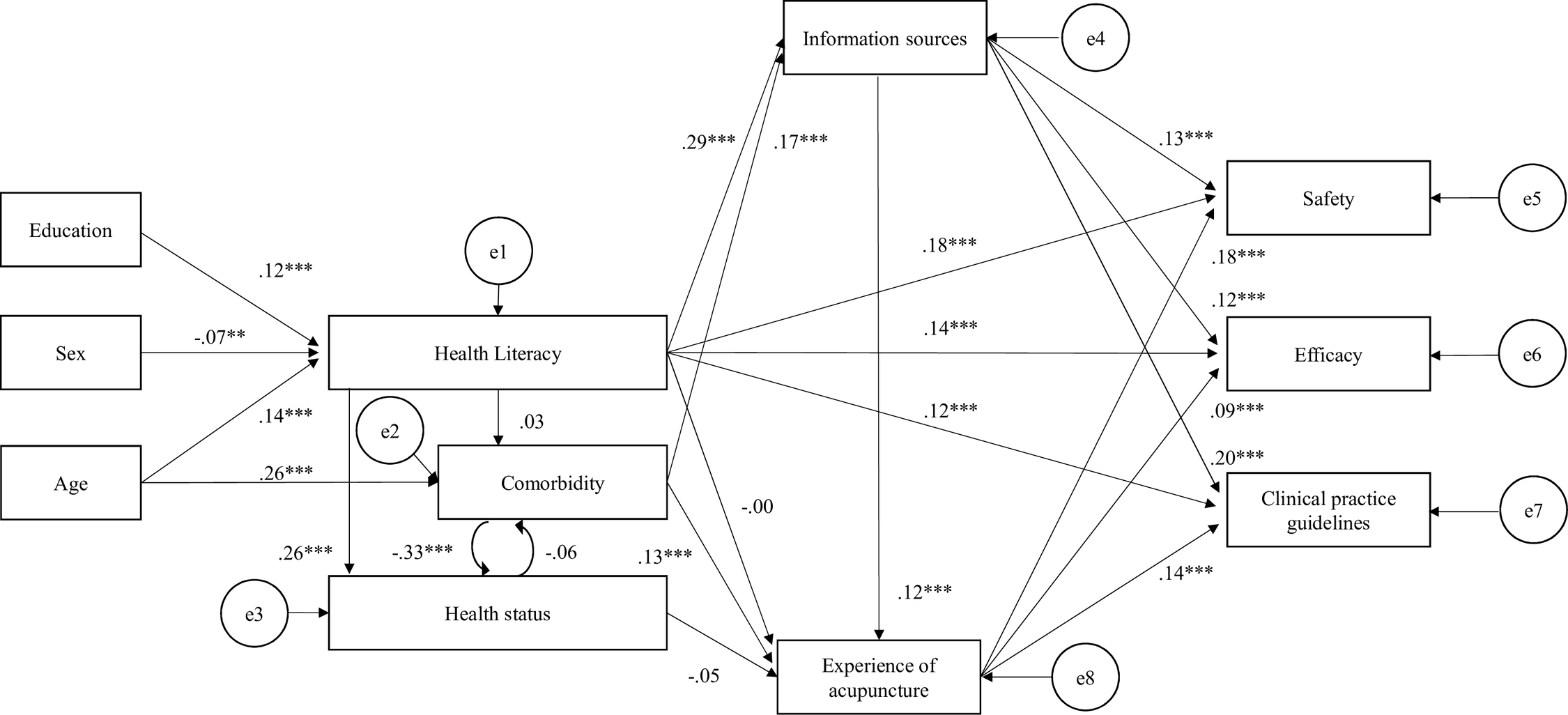

Supplement: S1 Fig — Rectangles are the observed variables. The values on the single-headed arrows are standardized regression weights. Model fitness: CFI = 0.968, RMSEA = 0.038 (95% CI 0.030–0.048). *p < 0.05, **p < 0.01, ***p < 0.001. (TIF) [file pone.0292729.s005.tif]
