# Supplementary material for: Relationship between health literacy and attitudes toward acupuncture: A web-based cross-sectional survey with a panel of Japanese residents
Source: PLoS One. 2023 Oct 20;18(10):e0292729. doi: 10.1371/journal.pone.0292729 (PMC10588898; doi:10.1371/journal.pone.0292729)
Supplement: Attachment — Submitted filename: Response_to_Reviewers.docx [file pone.0292729.s006.docx]

| **Journal Requirements** | | **Response** |
| --- | --- | --- |
| 1. Please ensure that your manuscript meets PLOS ONE's style requirements, including those for file naming. The PLOS ONE style templates can be found at <https://journals.plos.org/plosone/s/file?id=wjVg/PLOSOne_formatting_sample_main_body.pdf> and <https://journals.plos.org/plosone/s/file?id=ba62/PLOSOne_formatting_sample_title_authors_affiliations.pdf> | | The manuscript text, tables and figures were modified to meet PLOS ONE’s style requirements. |
| 2. Please provide additional details regarding participant consent. In the ethics statement in the Methods and online submission information, please ensure that you have specified (1) whether consent was informed and (2) what type you obtained (for instance, written or verbal, and if verbal, how it was documented and witnessed). If your study included minors, state whether you obtained consent from parents or guardians. If the need for consent was waived by the ethics committee, please include this information.  If you are reporting a retrospective study of medical records or archived samples, please ensure that you have discussed whether all data were fully anonymized before you accessed them and/or whether the IRB or ethics committee waived the requirement for informed consent. If patients provided informed written consent to have data from their medical records used in research, please include this information | | We added some phrases to specify that content was informed and written document on the web was used for obtaining informed consent.  **Ethical statement**  The study protocol was approved by the Ethics Committee of Morinomiya University of Medical Sciences (No. 2019-100). Regarding the protection of personal information, we confirmed that the internet research company, Mellinks, obtained PrivacyMark [~~21~~29]. The research panel members were informed of the objective, content, approximate time required for response, and protection policy of personal information, and that participation was not forced prior to starting the questionnaire. When the panel members agreed to become respondents with a written document on the web screen, they clicked on an agreement button, which started the questionnaire survey. The authors had no special access privileges to the data that could identify individual respondents.  (Lines 208–217) |
| 3. We note that you have stated that you will provide repository information for your data at acceptance. Should your manuscript be accepted for publication, we will hold it until you provide the relevant accession numbers or DOIs necessary to access your data. If you wish to make changes to your Data Availability statement, please describe these changes in your cover letter and we will update your Data Availability statement to reflect the information you provide. | | We do not wish to make changes to our Data Availability statement. |
| **Additional editor comments** | **Response to editor** | **How we revised the manuscript** |
| Please use an adequate reporting guides for your study: e.g., CHERRIES for online surveys and Todd 2017 (doi: 10.4236/psych.2017.89086) for SEM. | In accordance with the editor’s advice, we followed the reporting guidelines of CHERRIES and the article by Todd G Morrison et al. | We uploaded the filled checklist tables of these guidelines as “Supporting information” files.  We also revised the text as follows:  **Materials and methods**  We referred to the STROBE checklist [21], CHERRIES checklist [22], and reporting guidelines for SEM (Structural Equation Modelling) suggested by Morrison et al [23] when reporting the current study (S1–S3 Tables).  (Lines 98–100)  **“Study populations and procedures” in Materials and methods**  ~~From the database, 160 men and 160 women were selected from the panel of both sexes in each age group (20s (20–29 years old), 30s (30–39), 40s (40–49), 50s (50–59) and 60s (60–69)). Thus, a total of 1,600 participants living in Japan were included in this study.~~  This was a closed, panel-based survey that required registration for eligible respondents in order to be able to complete it. It was conducted on the Mellinks Web site for monitors registered with Mellinks' affiliated research firm. Prior to the questionnaire survey, approximately 40,000 potential survey monitors were randomly selected and contacted via e-mail or web notification with the survey protocol. The protocol notice provided an overview of the study design, including the name of the study investigator, on a Web screen, and confirmed the willingness of these potential respondents to participate in the study. Subjects who completed this voluntarily conducted survey were compensated, but the exact amount of compensation to participants was not disclosed by the research firm that was affiliated with Mellinks. Incidentally, the typical compensation for such surveys ranges from a few yen to 10 yen for the preliminary survey and from 50 yen to 100 yen for the actual survey.  Those who agreed to participate in this study were asked to provide their educational attainment and occupation, and these responses completed the preliminary survey. Next, Mellinks notified the consenting participants via e-mail or web-based invitation, and then the survey was conducted. All 11 questions were displayed on a single web page, and subjects were asked to answer all of them. To prevent missing data, this question session was designed not to be completed if there were unanswered questions. Once the participants had answered all the questions, they were asked to review their answers again, and they were allowed to revise them, if necessary. The survey targeted 160 men and 160 women in each age group (20s (20–29 years old), 30s (30–39), 40s (40–49), 50s (50–59) and 60s (60–69)).  Thus, a total of 1,600 participants living in Japan were included in this study. When 160 respondents in each age and sex group responded, the respective quota was closed. Responses were tabulated automatically.  (Lines 106–134)  **“Development of questionnaire”** **in Materials and methods**  Based on the inconvenience pointed out by several of those staff members, we carried out improvements to the questionnaire and a final version was completed. Then, the completed questionnaire was arranged by Mellinks for the Web survey. We checked the usability of the survey screen before it was released to the participants.  (Lines 159–162)  **“Data analysis” in Materials and method**  For this, first we performed a path analysis to test the hypothesis model established based on the ~~above cross-tabulation,~~results of above cross-tabulation and previous studies suggesting a relationship between health literacy and several factors, such as sociodemographic characteristics or the number of information sources [8], then trimmed non-significant paths to reach a final model.  (Lines 194–198)  All statistical analyses were performed using Jamovi Version 2.3.0 software [~~20].~~28]. The analysis was conducted without weighting the sample.  (Lines 202–203)  **Results**  The number of those who agreed to participate at the pre-survey stage was 3,292. Among them, the collection of responses continued until the number of respondents reached 160 in each of the age and sex quotas, and a total of 1,600 responses were finally collected. Therefore, the completion rate was of 48.6%.  (Lines 221–224) |
| Please address the following issues concerning the data analysis:  - neither / not sure responses were categorised as acupuncture disapproval. However, this option may also indicate low familiarity with acupuncture, and naturally be associated with low health literacy. As such, the conclusions of the current analysis are potentially misleading. | Regarding answers to Questions 8 to 10 (S1 Table. 11-item questionnaire), we divided the respondents into two groups: those who recognized acupuncture positively and those who did not, and then compared both groups. Therefore, the group of respondents who did not recognize acupuncture positively included not only those who answered “strongly disagree” and “disagree”, but also “Neither/not sure.” After the editor pointed this issue out to us, we realized that it was inappropriate to call the latter group the "disapproval group". Accordingly, we revised the "acupuncture disapproval group" to "acupuncture disapproval/neutral group."  On the other hand, we do not believe that the responses of low familiarity with acupuncture are necessarily associated with low health literacy. | **“Data analysis” in Materials and methods**  For Q8–10 which assessed the participants’ attitude toward acupuncture, respondents were divided into two groups of affirmation on acupuncture based on each respondent’s 5-point ratings; a score of four or more was regarded as the acupuncture approval (AA) group and that of less than four as the acupuncture disapproval (~~AD) group~~/Neutral (AD/N) group.  (Lines 177–181)  **Table 5.**  (We changed “AD” to “AD/N”, and “Acupuncture disapproval” to “Acupuncture disapproval/Neutral”.)  (Lines 339–342) |
| - actual positive/negative experience with acupuncture may override the beliefs driven by health literacy. The authors should explore this possible effect and find ways to reflect in the analysis. | As the editor pointed out, positive/negative experiences with acupuncture may override the beliefs driven by health literacy. However, we did not ask about the positive/negative experiences of respondents who had received acupuncture treatment in the present survey. Therefore, we cannot explore this possible effect and reflect on the analysis. Accordingly, we mentioned those limitations in the “Limitations of this study” subsection of the Discussion section. | **“Limitations of this study” in Discussion**  Fifth, for the 25.4% of respondents who had received acupuncture in their lifetime, their attitudes toward this therapy might have been influenced by actual positive or negative experiences, rather than their health literacy. Detailed questions on how those who received acupuncture in the past felt about the treatment were not included in this survey. Sixth, the measurement of health literacy was based on self-report, which may introduce biases, such as recall bias and over- or underestimation.  (Lines 467–473) |
| - using health literacy as a continuous predictor may be more adequate than a dichotomised item. | We analyzed health literacy as dichotomized variable according to previous research that Ishikawa et al. conducted. This will allow future comparisons with the data of Ishikawa et al. and papers using the same classification (e.g., Yoshizawa Y, et al. Int J Environ Res Public Health 2021;19:394 regarding frailty).  On the other hand, we used continuous variables for health literacy in the path analysis. Because this was not stated clearly, we added the explanation to the “Data analysis” subsection of the Methods section. | **“Data analysis” in Materials and method**  Additionally, we performed a path analysis to examine the relationships among sociodemographic factors (sex, age, and educational attainment), health literacy, the number of information sources, health status, and experience and attitudes toward acupuncture among the participants. For health literacy, we used continuous variables, while dichotomized data were used for cross-tabulation.  (Lines 188–192) |
| The applied SEM was confirmatory SEM, which requires the advance statement of a hypothesis (e.g. pre-specified model), and fit indices then indicate whether the hypothesis is plausible. In the context of this research, I suggest considering if exploratory SEM (eg. pls-SEM) would be more adequate than CB-SEM. (e.g. Hair 2019, doi 10.1108/EBR-11-2018-0203) In the current form, the analysis of results is potentially misleading. | The reason we used path analysis as a confirmatory SEM in this study is that we were interested in the observed variables from the question responses. Our main interest in this study was to determine whether or not there is a direct relationship between the level of health literacy of the Japanese people and their attitude toward acupuncture. Since the necessary data were available as observed variables, we decided that the analysis in this study was a simple model based on observed variables and that a path analysis would provide sufficient information.  Since the results were not strongly associated with any of the factors and were not inconsistent with the cross-tabulations, our conclusions are understated. Therefore, we do not believe that serious misleading occurred as a result of the use of path analysis in this study.  We have added an explanation to the Introduction section to better clarify the original purpose of our study, along with a response to advice 1) by Reviewer #1. | **Introduction**  The World Health Organization (WHO) defines health literacy as cognitive and social skills that determine the motivation and ability of individuals to gain access to, understand, and use information in ways that help to promote and maintain ~~good health~~wellbeing [1]. Previous reviews suggest that low health literacy is associated with poorer health outcomes and higher costs [2–4]. In Japan, health literacy scales with good reliability and validity have already been developed or translated by ~~prior~~previous studies [5–7], however, Japanese health literacy is reported to be lower than that of Europeans [7]. It has also been reported that Japanese people with higher health literacy are more likely to obtain sufficient health information from multiple sources and, thus, are less likely to engage in risky habits such as smoking and regular drinking [8]. However, it is not known whether the health literacy of the Japanese people influences the use of healthcare measures for which the evidence is not well established. In the West, studies suggest that people with high health literacy tend to have higher rates of use of complementary therapies [9,10]. In Japan, one typical example of a health care modality with insufficiently established evidence that has been used by some percentage of the population is acupuncture therapy.  In Japan, it has been reported that approximately 5–7% of people receive acupuncture, one of the most popular traditional and complementary therapies, at least once a year [~~9,10]~~.11,12]. Existing survey reports suggest that Japanese users of acupuncture tend to have lower education levels [11,12] although some confounding factors such as age may exist. If the education level and health literacy were to be similar, then Japanese people with lower health literacy would be more likely to receive acupuncture treatment.  The WHO encourages its member states to provide individuals with reliable information on the benefits and risks of integrating traditional and complementary medicine into their healthcare system [13].  Recently, studies have found evidence of the clinical effectiveness and safety of acupuncture; albeit insufficient, and there are some positive conclusions regarding several health conditions such as chronic low back pain and headaches in the Cochrane Database of Systematic Reviews [~~11–13]. However, it is necessary for people to have sufficient health literacy to correctly recognize reliable information regarding acupuncture. The WHO encourages its member states to provide individuals with reliable information on the likely benefits and risks of integrating traditional and complementary medicine into their healthcare system [14].~~14–19]. In addition, several evidence-based clinical practice guidelines have been developed in Japan that include recommendations for acupuncture [20]. If Japanese people with high health literacy gain knowledge about clinical evidence from multiple sources of health information, as in previous studies [8], then it can be predicted that not only those with low education levels but also those with high health literacy, will be willing to receive acupuncture, at least for health conditions for which some evidence of efficacy has been shown. To date, no direct data have been presented to support this hypothesis, and the aforementioned survey reports [11,12] suggesting a relationship between education levels and access to acupuncture are already out-of-date, with the most recent dating back to 2006.  ~~Relevant~~Thus, relevant research on the percentage of Japanese people that have knowledge about current evidence of acupuncture and the way that their knowledge of acupuncture and health literacy is related to their behavior in choosing this therapy is scarce. ~~Thus,~~Accordingly in this study, we conducted a web-based questionnaire survey to address this gap in research.  (Lines 46–94) |
| **Reviewer’s comments**  **（Reviewer #1）** | **Response to reviewer** | **How we revised the manuscript** |
| 1) Intro section: It is too short overall - this section needs to be profound. It is vital to state the objective of the study clearly | Following the advice, we added some text to the Introduction section. In particular, as pointed out in Comment 2), we have described in detail the background on the association between health literacy and attitudes toward acupuncture. | **Introduction**  However, it is not known whether the health literacy of the Japanese people influences the use of healthcare measures for which the evidence is not well established. In the West, studies suggest that people with high health literacy tend to have higher rates of use of complementary therapies [9,10]. In Japan, one typical example of a health care modality with insufficiently established evidence that has been used by some percentage of the population is acupuncture therapy.  (Lines 56–61)  Existing survey reports suggest that Japanese users of acupuncture tend to have lower education levels [11,12] although some confounding factors such as age may exist. If the education level and health literacy were to be similar, then Japanese people with lower health literacy would be more likely to receive acupuncture treatment.  The WHO encourages its member states to provide individuals with reliable information on the benefits and risks of integrating traditional and complementary medicine into their healthcare system [13].  (Lines 64–71)  In addition, several evidence-based clinical practice guidelines have been developed in Japan that include recommendations for acupuncture [20]. If Japanese people with high health literacy gain knowledge about clinical evidence from multiple sources of health information, as in previous studies [8], then it can be predicted that not only those with low education levels but also those with high health literacy, will be willing to receive acupuncture, at least for health conditions for which some evidence of efficacy has been shown. To date, no direct data have been presented to support this hypothesis, and the aforementioned survey reports [11,12] suggesting a relationship between education levels and access to acupuncture are already out-of-date, with the most recent dating back to 2006.  ~~Relevant~~Thus, relevant research on the percentage of Japanese people that have knowledge about current evidence of acupuncture and the way that their knowledge of acupuncture and health literacy is related to their behavior in choosing this therapy is scarce. ~~Thus,~~Accordingly in this study, we conducted a web-based questionnaire survey to address this gap in research.  (Lines 79–94) |
| 2) There needs to be more information regarding the association between health literacy and attitudes toward acupuncture.-> line 62,63,64. I have seen various articles regarding health literacy and CAM; however, this is the first time linking health literacy to acupuncture. It is essential to mention why. Do people not use acupuncture due to health literacy in Japan? Is there an article or statistics associated with it? | Same as above.  As mentioned in the revised Introduction, there have been no studies evaluating the direct relationship between health literacy and acupuncture use among the Japanese. | (Same as above) |
| 3) Please include a web-link for Mellinks. | We added web-link URL for Mellinks. | **“Study populations and procedures” in Materials and methods**  For this study, we conducted a web-based cross-sectional questionnaire survey between January 27 and February 3, 2020. The survey was commissioned to Mellinks Co., Ltd~~. (~~., Tokyo, Japan~~),~~ (https://www.mellinks.co.jp/), an internet research company that has nationwide panels by age group.  (Lines 103–106) |
| 4) Please indicate how Mellinks gather panels in the Methods section. | We added details on how Mellinks gathered panels in the Methods section. | **“Study populations and procedures” in Materials and Methods**  This was a closed, panel-based survey that required registration for eligible respondents in order to be able to complete it. It was conducted on the Mellinks Web site for monitors registered with Mellinks' affiliated research firm. Prior to the questionnaire survey, approximately 40,000 potential survey monitors were randomly selected and contacted via e-mail or web notification with the survey protocol. The protocol notice provided an overview of the study design, including the name of the study investigator, on a Web screen, and confirmed the willingness of these potential respondents to participate in the study. Subjects who completed this voluntarily conducted survey were compensated, but the exact amount of compensation to participants was not disclosed by the research firm that was affiliated with Mellinks. Incidentally, the typical compensation for such surveys ranges from a few yen to 10 yen for the preliminary survey and from 50 yen to 100 yen for the actual survey.  Those who agreed to participate in this study were asked to provide their educational attainment and occupation, and these responses completed the preliminary survey. Next, Mellinks notified the consenting participants via e-mail or web-based invitation, and then the survey was conducted. All 11 questions were displayed on a single web page, and subjects were asked to answer all of them. To prevent missing data, this question session was designed not to be completed if there were unanswered questions. Once the participants had answered all the questions, they were asked to review their answers again, and they were allowed to revise them, if necessary. The survey targeted 160 men and 160 women in each age group (20s (20–29 years old), 30s (30–39), 40s (40–49), 50s (50–59) and 60s (60–69)).  Thus, a total of 1,600 participants living in Japan were included in this study. When 160 respondents in each age and sex group responded, the respective quota was closed. Responses were tabulated automatically.  (Lines 110–134) |
| 5) Please indicate any rewards given to the panel and clearly state how you have recruited panels step-by-step. | We added details on whether any rewards were given to the panel and clearly stated how Mellinks recruited panels. | (Same as above) |
| 6) Are there no dropouts from 1600 people? | This survey was not conducted in a way that would cause dropouts. The details of the process are described at the beginning of the revised Results section. | **Results**  The number of those who agreed to participate at the pre-survey stage was 3,292. Among them, the collection of responses continued until the number of respondents reached 160 in each of the age and sex quotas, and a total of 1,600 responses were finally collected. Therefore, the completion rate was of 48.6%.  (Lines 221–224) |
| 7) Are there any discrepancies among the university staff members in reviewing the survey questions? | Several university staff members pointed out slight inconveniences, though not so much as a discrepancy in the pre-test stage. Therefore, we took their comments into account and made some improvements to the questionnaire. Then we completed the final version. We have added a note about this in the "Development of questionnaire" subsection of the Materials and Methods section. | **“Development of questionnaire” in Materials and methods**  In addition to the above 11 questions, we created questions for basic information on sociodemographics such as sex, age, educational attainment, occupation, and residential area. The developed questionnaire draft was pre-tested using our university staff members who were not included in the study panel to make it easier to answer. Based on the inconvenience pointed out by several of those staff members, we carried out improvements to the questionnaire and a final version was completed.  (Lines 155–162) |
| 8) Have you thought of performing a logistic regression analysis? | Initially, we considered using logistic regression analysis. However, we decided to use path analysis because logistic regression analysis requires a step-by-step examination, whereas path analysis allows us to examine the relationships among multiple variables simultaneously. | (No change was made) |
| **Reviewer’s comments**  **（Reviewer #2）** | **Response to reviewer** | **How we revised the manuscript** |
| To my understanding, the research question of this study is twofold. Firstly, this study examines the relationship between health literacy and the choice of acupuncture. It also aims to understand the extent to which the respondents' decision to use acupuncture depends on their knowledge and understanding of reliable sources. It also gives insight of the degree of health literacy of the Japanese population (based on a not representative sample). Other studies have been done on the relationship between the use of alternative and complementary medicine and health literacy, including those conducted among Japanese population. The novelty of the study is that it is the first to examine the relationship between the attitude to use acupuncture and health literacy among Japanese respondents. Narrowing the scope of complementary treatments to acupuncture could be justified by, that acupuncture is more widespread among the Japanese population than elsewhere in the world, so it may be relevant to study it in the context of health literacy. | We appreciate the careful and accurate summary of the features and novelty of this study by Reviewer #2. |  |
| I would suggest that the results of previous research on a similar topic also be mentioned in the introduction. | Following the suggestion given by Reviewer #2, we mentioned some previous studies indicating the relationship between health literacy and the use of complementary therapies, as well as those between education level and the use of acupuncture in the Introduction section. | **Introduction**  In the West, studies suggest that people with high health literacy tend to have higher rates of use of complementary therapies [9,10].  (Lines 57–59)    Existing survey reports suggest that Japanese users of acupuncture tend to have lower education levels [11,12] although some confounding factors such as age may exist.  (Lines 64–66) |
| To measure health literacy, they used a 5-item questionnaire developed by Ishikawa et al. The justification for the use of that scale seems incomplete. The authors have referred to several known measures, choosing one that has been piloted on a small sample size in a small circle (office workers). In this case, I think it is crucial to explain and justify the choice of the scale. | Indeed, the health literacy scale by Ishikawa et al. was developed in a small pilot study with 190 office workers. However, there were several advantages to using it. First, at least the internal reliability of the scale has been evaluated and warranted (Cronbach’s α = 0.86). Second, it consists of only five questions, making it compact and user-friendly for respondents. Third, it was subsequently used in some surveys targeting the Japanese population with a bigger sample (n=713 [Ishikawa 2016], n=1,002 [Goto 2019]).  We have added these reasons in the “Development of questionnaire” subsection in the Material and Methods section. | **“Development of questionnaire” in Materials and methods**  We developed a questionnaire comprising four categories (~~S1~~S4 Table): health status (Q1, 2), health literacy (Q3, 4), experience of receiving acupuncture (Q5, 6), and recognition and choice behavior regarding acupuncture (Q7–11). Regarding the health literacy measurement of Q3, we used a 5-item questionnaire developed by Ishikawa et al. [5]. Each item was rated on a 5-point scale, ranging from 1 (strongly disagree) to 5 (strongly agree). The reasons we used the scale by Ishikawa et al. [5], even though it was developed through a pilot study, are as follows: the internal reliability of the scale has been evaluated and warranted (Cronbach’s α = 0.86); it consists of only five questions, making it compact and user-friendly for respondents; it was subsequently used in some surveys targeting a bigger sample of the Japanese population [24,25].  (Lines 137–146) |
| The authors developed category variables from the health literacy scale scores. Respondents who scored above the group median were classified as high health literate, and those who scored below the group median were classified as low health literate. I think this is a good solution, but I would emphasise in the results that high and low health literacy should be understood in relation to the scores of the sample. | As pointed out later by the same reviewer, the sample in this survey did not match the Japanese population distribution. In the text, we have already noted that the number of respondents in each age group was not proportional to the population structure of Japan, but we have further stressed that the present results were obtained from a panel stratified by age and sex in the Conclusion section.  We also added a phrase to the title for readers to understand that this was a survey using a panel of respondents living in Japan. | **Title**  Japanese people’s health literacy and their attitudes toward acupuncture: A web-based cross-sectional survey with a panel of residents in Japan  **Conclusions**  In this study, which asked a panel of 1,600 respondents stratified by age and sex, we found that age, educational attainment, and sex might be associated with the health literacy of Japanese people, and older, educated, female participants tended to have higher health literacy.  (Lines 480–483) |
| Cross tabulation and chi-squares were used to examine the relationships between clusters, which are the appropriate statistical methods for the sample, and their results were reported in a clear and transparent way. | Thank you for the good evaluation regarding our statistical methods. |  |
| All scales used were based on self-report, a potential weakness of the study that is not addressed in the weaknesses section or in the description of the measurement scales. | We agree with the opinion of Reviewer #2. Therefore, we have pointed out this issue in the subsection “Limitation of this study” of the Discussion section. | **“Limitations of this study” in Discussion**  Sixth, the measurement of health literacy was based on self-report, which may introduce biases, such as recall bias and over- or underestimation.  (Lines 471–473) |
| For the descriptive statistical analysis of the data I would suggest the use of visualization tools (for example pie charts for describing the percentages of people self-perceived to be healthy, not healthy or for example the use of histograms to represent HL points distribution. | As Reviewer #2 suggested, we tentatively drew graphs to assess their usefulness. However, we found the self-perceived health status data to be too simple for a pie chart. We also found the health literacy data to be too complex to make a histogram, and the associations among the age group, sex, and high/low health literacy to be more difficult to comprehend than in a table. Therefore, we decided not to proceed with the suggested use of visualization tools. | (No change was made) |
| Both the conclusion and the title of the paper refer to a study of the attitudes of Japanese people, but the sample was not representative (as the authors pointed out transparently), so I think it would be more accurate to limit the conclusions to the sample rather than to Japanese people. | To limit the conclusions to the sample, we have added a phrase stressing that the present results were obtained from a panel stratified by age and sex in Title, “Limitations of this study” in Discussion and Conclusion. | **Title**  Japanese people’s health literacy and their attitudes toward acupuncture: A web-based cross-sectional survey with a panel of residents in Japan  **Limitations of this study**  Despite these limitations, this is the first study to assess the relationship between health literacy ~~of Japanese people in relation to their~~and attitudes toward acupuncture, and it provides useful suggestions for the future of health education.  (Lines 474–476)  **Conclusions**  In this study, which asked a panel of 1,600 respondents stratified by age and sex, we found that age, educational attainment, and sex might be associated with the health literacy of Japanese people, and older, educated, female participants tended to have higher health literacy. Furthermore, it can be deduced that consulting medical doctors and reading newspapers may have influenced the level of participants’ health literacy. Although Japanese people with higher health literacy are more likely to perceive acupuncture positively, they do not necessarily have sufficient knowledge of the clinical evidence of acupuncture, and their decision to receive acupuncture may be more dependent on personal narratives from their family and acquaintances rather than clinical research evidence.  (Lines 480–489) |
| One recommendation regarding the format is that I would suggest the authors formatting the tables in a standard format. | Thank you for the suggestion. We modified tables to match the standard format.  While we were rechecking all the tables and figures, we noticed that the legends for Figures 1 and 2 were not all showing. Therefore, we corrected the legends for Figures 1 and 2. | **Tables 1, 2, 3, 4 and 5**  Table rules were modified to conform to the standard format.  **Figures 1 and 2**  Legends were corrected. |
| Overall, in my opinion the research question has been answered with appropriate statistical tests and the quality of the reporting is good. I therefore propose the acceptance of the paper with minor adjustments. | We appreciate the favorable evaluation of our research paper. |  |
| **Additional revision for some parts of the manuscript**  **that were not suggested by the editor or reviewers.** | | **How we revised the manuscript** |
| To clarify how we classified the respondents who answered Q8 into two groups (acupuncture approval and acupuncture disapproval/neutral group), we added a sentence to explain the detail. | | **“Data Analysis” in Materials and methods**  In Q8, the division of the respondents into two groups (AA and AD/N group) depended on whether their median score for six symptoms was four or higher.  (Lines 181–183) |
