# Supplementary material for: Relationship between health literacy and attitudes toward acupuncture: A web-based cross-sectional survey with a panel of Japanese residents
Source: PLoS One. 2023 Oct 20;18(10):e0292729. doi: 10.1371/journal.pone.0292729 (PMC10588898; doi:10.1371/journal.pone.0292729)
Supplement: Attachment — Submitted filename: Response_to_Reviewers.docx [file pone.0292729.s007.docx]

| **Reviewer’s comments**  **（Reviewer #1）** | **Response to reviewer** | **How we revised the manuscript** |
| --- | --- | --- |
| All comments have been addressed; however, I would like to stress out that this manuscript needs to get professional editing and proofreading services. | Thank you for your comment. Our manuscript has already been reviewed and revised by Editage, but the previous revision process may have caused some unnatural English sentences. We asked Editage to re-check the entire manuscript again this time. We have attached a certificate of editing for this revised manuscript. | [*Title*]  Japanese people’s health literacy and their attitudes toward acupuncture: A web-based cross-sectional survey with a panel of Japanese residents ~~in Japan~~  (Lines 1–2)  [*Abstract*]  The relationship between health literacy of Japanese people~~’s health literacy~~, their attitudes toward acupuncture, and their behavior in choosing this therapy is currently unclear. Therefore, for this study, we conducted a web-based survey to address this unknown relationship.  A questionnaire comprising four categories (health status, health literacy, previous acupuncture experience, and attitudes toward acupuncture) was administered to 1,600 Japanese participants. For this study, we performed cross-tabulation and path analysis to examine the relationship between each questionnaire item. The mean score of participants’ health literacy was 3.41 (SD = 0.74), and older, educated, female participants tended to have higher health literacy. ~~Acupuncture was~~ The respondents perceived ~~as~~ acupuncture to be effective for chronic low back pain, tension-type headache, and knee pain due to osteoarthritis ~~by~~ (40.0%, 38.7%, and 21.8% ~~of respondents,%~~, respectively~~. In contrast~~). Contrastingly, acupuncture was perceived as far less effective for postoperative nausea/vomiting and prostatitis symptoms (8.4% and 8.7%, respectively). Of the total study respondents, 34.4% reported that they would try acupuncture only if recommended by clinical practice guidelines, and 35.6% agreed that acupuncture is safe. The path analysis showed that attitudes toward acupuncture were significantly influenced by the participants’ health literacy, ~~the~~ number of information sources, and previous acupuncture experience. However, it was also found that experience with acupuncture was not directly associated with health literacy.  Although the Japanese population with higher health literacy is more likely to perceive acupuncture positively, they do not necessarily have sufficient relevant knowledge of the clinical evidence ~~on acupuncture, and thus~~. Therefore, their decision to receive acupuncture may be more dependent on personal narratives rather than clinical evidence. Thus, ~~the~~ future challenges lie in individual education of the population on how to choose a reliable health information source, and organizational efforts to provide more reliable health information.  (Lines 19–44)  [1st paragraph of *Introduction*]  The World Health Organization (WHO) defines health literacy as cognitive and social skills ~~that~~ which determine the motivation and ability of individuals to gain access to, understand, and use information in ways ~~that help to~~ which promote and maintain ~~wellbeing~~ good health [1]. Previous ~~reviews~~ studies suggest that low health literacy is associated with poorer health outcomes and higher costs [2–4]. In Japan, health literacy scales with good reliability and validity have already been developed or translated by previous studies [5–7~~], however,~~]. However, a prior study has found that Japanese health literacy is ~~reported to be~~ lower than that of Europeans [7]. It has also been reported that Japanese people with higher health literacy are more likely to obtain sufficient health information from multiple sources and, thus, are less likely to engage in risky habits ~~such as~~like smoking and regular drinking [8]. However, it is ~~not known~~still unknown whether the health literacy of the Japanese people influences the use of healthcare measures for which the evidence is not well established as yet. In the West, studies suggest that people with high health literacy tend to ~~have higher rates of use of~~use complementary therapies more [9,10]. In Japan, one typical example of a ~~health care~~healthcare modality with insufficiently established evidence that has been used by some percentage of the population is acupuncture therapy.  (Lines 46–61)  [*Materials and methods*]  We referred to the STROBE checklist [21], CHERRIES checklist [22], and reporting guidelines for SEM (~~Structural Equation Modelling~~ structural equation modeling) suggested by Morrison et al [23] when reporting the current study (S1–S3 Tables).  (Lines 104–106)  [“Study populations and procedures” in *Materials and methods*]  This was a closed, panel-based survey that required registration for eligible respondents ~~in order~~ to be able to complete it. It was conducted on the Mellinks ~~Web site~~ website for monitors registered with Mellinks~~'~~’ affiliated research firm. Prior to the questionnaire survey, approximately 40,000 potential survey monitors were randomly selected and contacted via e-mail or web notification with the survey protocol. The protocol notice provided an overview of the study design, including the name of the study investigator, on a ~~Web screen~~ webpage, and confirmed the participants’ willingness ~~of these potential respondents~~ to participate in the study. ~~Subjects~~ Those who completed this ~~voluntarily conducted~~ survey were compensated~~, but~~; however, the exact amount of compensation ~~to participants~~ was not disclosed by the research firm that was affiliated with Mellinks. Incidentally, the typical compensation for such surveys ranges from a few yen to 10 yen for the preliminary survey and from 50 yen to 100 yen for the actual survey.  Those who agreed to participate in this study were asked to provide their educational attainment and occupation, ~~and these responses completed~~ which indicated the completion of the preliminary survey. ~~Next~~ Thereafter, Mellinks notified the consenting participants via e-mail or web-based invitation, ~~and then~~before commencing the survey ~~was conducted.~~. All 11 questions were displayed on a single web page, and ~~subjects~~ the participants were asked to answer all ~~of them~~. To prevent missing data, this question session was designed ~~not to~~ in a manner that it will be ~~completed~~ considered as incomplete if there were unanswered questions. Once the participants had answered all the questions, they were asked to review their answers again~~,~~ and ~~they~~ were allowed to revise them, if ~~necessary~~ required. The survey targeted 160 men and 160 women in each age group (20s (20–29 years ~~old~~), 30s (30–39 years), 40s (40–49 years), 50s (50–59 years) and 60s (60–69~~)).~~ years)).  (Lines 113–134)  [“Development of questionnaire” in *Materials and methods*]  ~~In addition to~~ Along with the ~~above~~ abovementioned 11 questions, we created questions for basic information on ~~sociodemographics such as~~ sociodemographic attributes like sex, age, educational attainment, occupation, and residential area. The developed questionnaire draft was pre-tested using our university staff members who were not included in the study panel to make it easier to answer. Based on the inconvenience pointed out by several of th~~os~~e staff members, we ~~carried out improvements to~~ improved the questionnaire ~~and a~~ to arrive at the final version ~~was completed. Then.~~ Thereafter, the completed questionnaire was arranged by Mellinks for the ~~W~~web survey. We checked the usability of the survey screen before releasing it ~~was released~~ to the participants.  (Lines 161–168)  [“Data analysis” in *Materials and methods*]  ~~Descriptive~~For data analysis, descriptive statistics, ~~such as~~ like frequencies, means, and cross-tabulations, were calculated. Based on responses to Q3 (health literacy measures), respondents were divided into two groups of health literacy according to the median score of five items (5-point rating each): a median score of four or more was regarded as the higher health literacy (HHL) group, and that of less than four, as the lower health literacy (LHL) group ~~(~~[5~~).~~].  (Lines 178–183)  Using these classifications, we assessed the interrelationships among sex, health literacy, experience of acupuncture, and attitudes toward acupuncture through cross-tabulation. Pearson’s chi-squared test was used for these analyses. Odds ratios and 95% confidence intervals ~~a~~were shown as effect sizes.  (Lines 190–193)  For this, first we performed a path analysis to test the hypothesis model established based on the results of above cross-tabulation and previous studies suggesting a relationship between health literacy and several factors, ~~such as~~like sociodemographic characteristics or the number of information sources [8], then trimmed non-significant paths to reach a final model. The fitness of the model was evaluated using comparative fit index (CFI) and root mean square error of approximation (RMSEA). If the CFI was larger than 0.95 and the RMSEA was < 0.05, the model was considered to be acceptable [27].  All statistical analyses were performed using the software, Jamovi Version 2.3.0 ~~software~~ [28]. The analysis was conducted without weighting the sample. For path analysis, we used Jamovi’s modules of PATH ANALYSIS 0.8.0. The significance level was set at p < 0.05.  (Lines 200–211)  [“Ethical statement” in *Materials and method*]  The study protocol was approved by the Ethics Committee of Morinomiya University of Medical Sciences (No. 2019-100). Regarding the protection of personal information, we confirmed that the internet research company, Mellinks, obtained PrivacyMark [29]. The research panel members were informed of the objective, content, approximate time required for response, and protection policy of personal information, and the fact that participation was not forced prior to starting the questionnaire. When the panel members agreed to become respondents with a written document on the web ~~screen~~page, they clicked on an agreement button, which started the questionnaire survey. The authors had no special access privileges to the data that could identify individual respondents.  (Lines 214–223)  [*Results*]  The total number of those who agreed to participate at the pre-survey stage was 3,292. Among them, the collection of responses continued until the number of respondents reached 160 in each of the age and sex quotas, and a total of 1,600 responses were finally collected. Therefore, the completion rate was ~~of~~ 48.6%.  (Lines 227–230)  [“Sociodemographic characteristics of respondents” in *Results*]  The characteristics of the respondents in terms of sex, age group, educational attainment, occupation, and residential area are shown in Table 1. Among the male respondents, university graduates and office workers were the ~~most common~~ majority in the sample, while high school graduates and houseworkers were the ~~most common~~ majority among females. It is noteworthy that the number of respondents in each age group was 160 for both males and females and was not proportional to the population structure of Japan.  (Lines 233–238)  [“Health status (Q1, 2)” in *Results*]  In total, the proportion of those who thought they were healthy (“agree” + “strongly agree”), unhealthy (“disagree” + “strongly disagree”) and “neither” was 45.3%, 28.9% and 25.9%, respectively. This trend ~~was~~ could also ~~similar when focusing on~~ be seen in the 60s age group (46.6%, 30.0%, and 23.4%, respectively).  (Lines 243–246)  [“Health literacy (Q3, 4)” in *Results*]  The mean score for the five items in Q3 (each item scored on a 5-point scale) was 3.41 (SD = 0.74), which tended to be higher ~~as the~~with increasing age group ~~increased~~. Divided into two groups (HHL ≥ 4, LHL < 4) according to the median score of five items, the number of respondents with HHL was 797 (49.8%) and those with LHL was 803 (50.2%). In the 20s age group, the proportion of male respondents with HHL tended to be small (Table 2).  ~~As sources of health information asked in Q4, television was the most common source of information followed by internet and blogs in all age groups, except for those in their 40s. Another feature was that the use of social networking sites (SNS; Twitter, Facebook, Instagram) for obtaining relevant information decreased as the age group increased while medical doctors and newspapers showed the opposite trend (Fig 1).~~  ~~Table 3 shows the relationship between the level of health literacy (HHL vs. LHL) and the use of each source of health information. Significant differences in the use of health information sources were found according to the level of health literacy, except for radio and SNS. There was a particularly large odds ratio between HHL and LHL regarding the use of books, internet, and newspapers (3.49, 3.04, and 2.72, respectively).~~  (Lines 255–260)  [“Experience of acupuncture (Q5, 6)” in *Results*]  For the questions regarding experience of acupuncture, 61 respondents (3.8 %) answered that they were currently receiving acupuncture treatment, and 70 (4.4%) ~~respondents~~ answered that they were “not currently receiving but have received within the past year”. Therefore, the percentage of annual use of acupuncture by the Japanese people was 8.2% (95% CI: 6.9–9.6). The percentage of those who had received acupuncture at least once in the past, that is, lifetime use, was 25.4% (95% CI: 23.3–27.6).  Among those who ~~answered that~~ responded to “have never received” (1,194 respondents), the most common reason was ~~“~~because ~~I~~ they “did not feel the necessity” (52.3%), followed by “because I was healthy” (25.0%), “because I thought it would be expensive” (15.6%), and “because acupuncture seemed painful” (14.2%). In the female respondents, the third most common reason was “because acupuncture seemed painful” while this answer was the sixth most common in the male respondents (Table 4).  (Lines 289–300)  [“Information source, knowledge, and attitudes on acupuncture (Q7–11)” in *Results*]  Regarding the source of information for deciding whether to receive acupuncture (Q7), the most common answer among all respondents was family, friends, and acquaintances (30.3%), followed by internet and blogs (except for SNS) (29.7%), and medical doctors (27.0%). The respondents were most likely to use the internet and blogs in their 20s to 40s~~;~~. ~~i~~In their 50s, they would essentially rely on family, friends, and acquaintances, and on medical doctors in their 60s (Fig 2).  Fig 2. Sources of information that the respondents rely on deciding whether to choose acupuncture.  Respondents could choose more than one resource option; hence, this figure includes multiple answers. SNS: Social networking sites  * Except for SNS  The most common condition for which acupuncture was recognized as effective among the six conditions listed in Q8 was chronic low back pain (40.0%; the percentages of “agree” + “strongly agree”), followed by tension-type headache (38.7%), and knee pain due to osteoarthritis (21.8%). ~~In contrast~~ Contrastingly, acupuncture was considered far less effective for postoperative nausea/vomiting and prostatitis symptoms (8.4% and 8.7%, respectively). Note that the most common answer was “neither/not sure” for all six conditions listed in Fig 3.  Fig 3. Degree to which respondents recognize the effectiveness of acupuncture for each condition.  * Tension-type headache (associated with neck and shoulder stiffness)  ** Postoperative nausea and vomiting  For the question “If acupuncture is recommended in clinical practice guidelines about your conditions or diseases, are you going to try acupuncture?” 34.4% of the respondents answered “agree” or “strongly agree,” 22.6% answered “disagree” or “strongly disagree,” and 43.0% chose “neither~~”.~~.” As to the question on the safety of acupuncture (Q10), 35.6% answered “agree” or “strongly agree”, 12.6% answered “disagree” or “strongly disagree”, and 51.8% chose “neither”.  ~~Fig 2. Sources of information that the respondents rely on deciding whether to choose acupuncture.~~  ~~Respondents could choose more than one resource option; hence, this figure includes multiple answers. SNS: Social networking sites~~  ~~* Except for SNS~~  ~~Fig 3. Degree to which respondents recognize the effectiveness of acupuncture for each condition~~  ~~* Tension-type headache (associated with neck and shoulder stiffness)~~  ~~** Postoperative nausea and vomiting~~  (Lines 306–337)  [“Path analysis” in *Results*]  Confirming that the fitness indices of the hypothesis model (S5 Fig) were adequate (CFI = 0.968 and RMSEA = 0.038 [95% CI: 0.030–0.048]), the final model was developed after removing the non-significant path, as shown in Fig 4. It was noted that the model fitness indices improved~~:~~ [CFI = 0.978 and RMSEA = 0.031 (95% CI: 0.022–0.040~~).~~)].  (Lines 357–361)  [*Discussion*]  The annual use of acupuncture was 8.2% (95% CI: 6.9–9.6), which was slightly higher than that reported in previous studies, being 6.7% (95% CI: 5.2–8.2) in 2000 [11] and 6.7% (95% CI: 5.2–8.3) in 2005 [12]. Since our survey panel is not proportional to the population of Japan, it is difficult to directly compare previous studies with that of ours. Nevertheless, it is obvious that the annual use of acupuncture in Japan is higher than that in almost all Western countries [30,31].  In this study, based in Japan, where a relatively larger proportion of the population receives acupuncture than in the West, we focused on whether there is any relationship between health literacy and the choice of acupuncture, along with determining the degree of health literacy of the Japanese population. ~~In other words~~ Thus, this study also aimed to ~~know~~ understand whether the Japanese public are making evidence-based choices for acupuncture, the efficacy of which is controversial.  (Lines 382–394)  [“Acupuncture and health literacy” in *Discussion*]  This study found that relatively more respondents recognized that acupuncture was effective for chronic low back pain and tension-type headache. However, less than 10% of them thought that this therapy would be effective for alleviating ~~postoperative~~ post-operative nausea/vomiting and prostatitis symptoms, indicating a discrepancy between the evidence presented by Cochrane reviews and public perception.  ~~The~~This study’s findings ~~of this study~~ are not limited to Japan. Previous studies conducted in other countries have also shown similar results. For example, only 15.5% and 10% of respondents in Australia and China, respectively, thought that acupuncture could reduce nausea and vomiting [36,37].  Based on the path analysis (Fig 4), it was suggested that Japanese people with higher health literacy are more likely to perceive acupuncture positively. Nevertheless, we could not find a direct relationship between health literacy and acupuncture experience (Table 5, Fig 4). This may be because even respondents with higher health literacy were not provided with sufficient evidence for acupuncture. ~~Not~~ Thus, it can be said that utilizing potentially effective healthcare measures without being informed about the evidence would be a loss of benefit to the people concerned. Therefore, sufficient evidence and higher health literacy are needed to promote and maintain a better health.  (Lines 426–442)  [“Healthcare decision-making and health information source” in *Discussion*]  With respect to the level of evidence, clinical practice guidelines would be an ideal health information source if they were well-produced and trustworthy [38]. In this study, only 34.4% of the respondents chose “agree” or “strongly agree” to the question “if acupuncture is recommended in clinical practice guidelines about your conditions or diseases, are you going to try acupuncture?”~~,~~ and 43.0% chose “neither~~”. In view of this result~~.” Accordingly, we suspect that many respondents did not understand the significance of clinical practice guidelines.  (Lines 454–460)  [“Limitations of this study” in *Discussion*]  Third, we did not collect data on the respondents’ income, which may have influenced the path analysis, particularly in terms of education, health literacy, and experience of acupuncture.  (Lines 474–476)  [Conclusions]  In this study, ~~which asked~~ involving a panel of 1,600 respondents stratified by age and sex, we found that age, educational attainment, and sex might be associated with the health literacy of Japanese people~~, and older.~~ Older, educated, female participants tended to have higher health literacy.  (Lines 492–495) |
| **Reviewer’s comments**  **（Reviewer #3）** | **Response to reviewer** | **How we revised the manuscript** |
| This is an excellent study and the authors have revised the manuscript in response to comments made by other reviewers. However, the authors need to make further revisions and improvements to address the following points.  * The background needs to continue to be refined to explain the need for the study. | Thank you for your comment. We would like to thank the reviewers for their detailed evaluation of our manuscript.  We have once again revised and added relevant text so that the readers can understand the background as to why this survey study required to be conducted.  We also explained two different issues that may be potentially confusing: “health literacy and willingness to receive acupuncture” and “health literacy and actual experience of receiving acupuncture.” | [2nd paragraph of *Introduction*]  (Revised as below: see next cell)  [3rd paragraph of *Introduction*]  The WHO encourages its member states to provide individuals with reliable information on the benefits and risks of integrating traditional and complementary medicine into their healthcare system [13]. Recently, studies have found evidence of ~~the~~ clinical effectiveness and safety of acupuncture~~;~~. ~~a~~Albeit insufficient, ~~and~~ there are some positive conclusions regarding ~~acupuncture for~~ several health conditions ~~such as~~ like chronic low back pain and headaches in the Cochrane Database of Systematic Reviews [14–19]. ~~In addition~~Furthermore, several evidence-based clinical practice guidelines have been developed in Japan that include recommendations for acupuncture [20]. If the Japanese ~~people~~population with a high health literacy gain knowledge about clinical evidence from multiple sources of health information, as shown in a previous stud~~ies~~y [8], then it can be predicted that ~~not only those with low education levels but also those with high health literacy,~~ they will be willing to receive acupuncture, at least for health conditions for which some evidence of efficacy has been shown. To date, no direct data have been presented to support this hypothesis, and the aforementioned survey reports [11,12] ~~suggesting~~ also have not shown clear answers regarding a relationship between ~~education levels~~ people’s health literacy and ~~access to~~ experience of receiving acupuncture ~~are already out-of-date, with the most recent dating back to 2006.~~  (Lines 75–89)  [4th paragraph of *Introduction*]  Thus, relevant research on the percentage of Japanese people that have knowledge about current evidence of acupuncture ~~and the way that~~ is scarce. Furthermore, it is also relatively unknown how their knowledge of evidence on acupuncture and health literacy ~~is~~are related to their behavior in choosing this therapy ~~is scarce. Accordingly~~. Moreover, having a positive attitude toward a therapy based on health literacy may be different from actually receiving that therapy. Hence, if we take acupuncture as an example to clarify the characteristics of the Japanese people’s health literacy and their attitudes toward controversial treatments with insufficient evidence and actual experience, the key points of future specific educational policies on health literacy for the public would become much clearer. Thus, in this study, we conducted a web-based questionnaire survey to address th~~is~~e gap in relevant body of research.  (Lines 90–100) |
| *Page 5, Line 66-68: Why did the authors make this assumption? Based on the previous description, it is difficult to think of this assumption. | Thank you for your comment. On re-reading the references and writing again, we realized that we had somewhat confused health literacy and education levels, whose relationship is not necessarily clear at the pre-survey stage. Therefore, we have removed this sentence and tried to explain the link between people’s health literacy and their health behaviors about acupuncture that has been unclear. | [2nd paragraph of *Introduction*]  ~~In Japan, it~~It has been reported that in Japan, approximately 5–7% of people receive acupuncture, one of the most popular traditional and complementary therapies, at least once a year [11,12]. ~~Existing~~ Musculoskeletal problems were the most common condition for which acupuncture was used (approximately 80%), and referral by family or friends was the most common reason for people to receive acupuncture (approximately 60%) [12]. In around the 2000s, it was unknown how widespread information on the evidence for acupuncture was among the Japanese population; additionally, it was unclear how much their health literacy played a role in their decision to use acupuncture for their intended condition. Incidentally, existing survey reports suggest that Japanese users of acupuncture tend to have lower education levels [11,12~~] although some confounding factors such as age may exist. If the education level and health literacy were to be similar, then Japanese people with lower health literacy would be more likely to receive acupuncture treatment.~~], but these results were probably confounded by age factor because the older population were more likely to use acupuncture than the younger [12]. Moreover, the relationship between health literacy and education levels has been controversial among studies in Japan [7,8,35].  (Lines 62–74) |
| *Page 8, Line121-123: In the background of the study, the authors primarily wanted to address the correlation between health literacy (Education Level, line 76-84) and people's attitudes toward acupuncture, so why did the authors not group people according to education level but rather according to age? Here the authors need to provide an explanation and cite evidence to justify the need to do so. | Thank you for your comment. As noted above, we had confused health literacy with education level, but it was not clear that they were congruent. Therefore, we have deleted that section and rewritten it (please see the previous response).  What we primarily wanted to know was health literacy, and we sought to explore the relationship with education level by analyzing post-survey data. Therefore, we set the quotas by age and gender, as is common in this type of panel survey, rather than by education level, and we have not stated any particular reason for this. | (No change) |
| *Page 8, line 129: What were the main areas of pre-work or reference on which the questionnaire was developed? Who were the people involved in the development of the questionnaire? What are the backgrounds of each of these people? The authors need to elaborate. | Thank you for your comment. The questionnaire in this study was developed by researchers (YO and HY) who have conducted clinical and survey research on acupuncture and a statistician (NI) in the field of psychology. It was based on the paper by Ishikawa et al [5].  The pilot study of Ishikawa et al [5] to develop a questionnaire for health literacy assessment was conducted by questions to 190 male office workers during an annual health check-up. The scale items of the questionnaire draft were constructed to directly reflect the WHO definitions [5]. Researchers involved in this pilot study [5] were specialists in public health at Japanese universities, and in particular the first and last authors of the cited paper [5] have published many articles on health literacy.  We have added relevant brief information to the text about the background of the development of the questionnaire by Ishikawa et al, on which our questionnaire was based. | [“Development of questionnaire” in *Materials and methods*]  We developed a questionnaire comprising four categories (S4 Table): health status (Q1, 2), health literacy (Q3, 4), experience of receiving acupuncture (Q5, 6), and recognition and choice behavior regarding acupuncture (Q7–11). Regarding the health literacy measurement of Q3, we used a 5-item questionnaire developed by Ishikawa et al~~. [5].~~., who are considered to be specialists in public health who conducted a pilot study questioning 190 male office workers during an annual health check-up. The scale items of the questionnaire draft were constructed to directly reflect the WHO definitions [5]. Each item was rated on a 5-point scale, ranging from 1 (strongly disagree) to 5 (strongly agree). ~~The reasons we used the scale by Ishikawa et al. [5], even though it was developed through a pilot study, are as follows~~ We chose this scale [5] for this study because of the following reasons: the internal reliability of the scale has been evaluated and warranted (Cronbach’s α = 0.86); it ~~consists of~~ comprises only five questions, making it compact and user-friendly for respondents; it was subsequently used in some surveys targeting a bigger sample of the Japanese population [24,25].  (Lines 140–152) |
| *Page 13, line 215: Therefore, the completion rate was of 48.6%. A completion rate (effective rate) of 80% for the questionnaire would be considered feasible for implementation. However, 48.6% was reported here. Have the authors considered the reasons for this result? | Thank you for your comment. This is a panel-based study, and even the 3,292 participants in the pre-survey stage were survey monitors who were pre-registered with the research firm. This has already been described in the sub-section, “Study populations and procedures” in Materials and methods.  Thus, the completion rate is quite different from the response rate in surveys such as random-digit dialing. When all quotas of age and sex were filled (in total 1,600 respondents), the survey automatically ended. Thereafter, the completion rate (CHERRIES checklist [22]) was calculated to be 48.6%. The process for completing this survey is also described in “Study populations and procedures” in Materials and methods section. | (No change) |
|  | During the process of re-checking the manuscript, we noticed that there are some contradictions between the present results and previous studies regarding factors that influence health literacy. Accordingly, we amended the sentence describing the comparison with preceding reports in the Discussion section. | [“Health literacy of the Japanese people” in *Discussion*]  Generally, health literacy levels depend on the social, cultural, and demographic background ~~such as~~ like age, sex, educational attainment, race, marital status, employment status, income, and insurance status of the participants’ residential country [9,32–34]. In this study of Japanese people, we found that age, sex, and educational attainment were associated with health literacy (Fig 4). Specifically, older, educated, and female participants tended to have higher health literacy. These results are not necessarily consistent with those of previous studies conducted in Japan probably due to differences in sample population characteristics ~~except for a few contradictory results regarding the association with age~~ [5–8,35].  ~~Thus, w~~We speculate that the differences in information sources among age groups (medical doctors, newspapers, and SNS; Fig 1) may have some influence on the gradual increase in health literacy among age groups. ~~Those~~ Interestingly, it was found that those in their 60s obtained more information from television and the internet followed by medical doctors and newspapers. ~~In contrast~~ Contrastingly, those in their 20s obtained more information from SNS (Fig 1).  (Lines 397–410) |
