# Supplementary material for: Relationship between health literacy and attitudes toward acupuncture: A web-based cross-sectional survey with a panel of Japanese residents
Source: PLoS One. 2023 Oct 20;18(10):e0292729. doi: 10.1371/journal.pone.0292729 (PMC10588898; doi:10.1371/journal.pone.0292729)
Supplement: Attachment — Submitted filename: Response_to_Reviewers.docx [file pone.0292729.s008.docx]

| **Editor’s comments** | **Response to Editor** | **How we revised the manuscript** |
| --- | --- | --- |
| In the Introduction: The authors must expound more clearly on this study's precise objectives. It would be prudent to incorporate a more comprehensive presentation of studies concerning health literacy and acupuncture, not solely confined to Japan but also encompassing nations where acupuncture holds a notable prevalence. | Thank you for your comment. We stated the aim of the study clearly in the last paragraph of the Introduction section. Regarding the present status of research findings on the relationship between health literacy and acupuncture, we could not find the relevant studies outside Japan even after we broadened the scope to include the entire range of complementary therapies.  Accordingly, we added a sentence explaining that fact at the end of the 2nd paragraph of the Introduction section.  Additionally, we have reviewed and revised the entire Introduction section to provide a better understanding of the background of this study. | *[Introduction]*  According to The World Health Organization (WHO) definition, health literacy refers to cognitive and social skills which determine the motivation and ability of individuals to gain access to, understand and use information in ways which promote and maintain good health [1]. The WHO identifies health literacy as one of the three pillars of health promotion (good governance, healthy cities and health literacy) and recognizes it as a critical determinant of health [2]. Previous studies suggest that low health literacy is associated with poorer health outcomes and higher costs, such as more hospitalizations, greater use of emergency services and higher mortality rates in older people [3–5]. Studies including measurement of health literacy have been conducted, particularly in the United States, although the evidence on the relationship between health literacy and health disparities is still scarce and limited [6].  In Japan, health literacy scales with good reliability and validity have already been developed or translated by previous studies and Japanese people’s health literacy has been assessed using those scales [7–9]. It has been reported that Japanese people with higher health literacy are more likely to obtain sufficient health information from multiple sources and, thus, are less likely to engage in risky habits like smoking and regular drinking [10]. However, a survey has found that Japanese health literacy is relatively lower than that of Europeans [9]. The authors who conducted that survey speculate that this may be due in part to an insufficient primary health care system and difficulty accessing reliable health information, but no solid factors have been identified. Moreover, it is unknown whether the health literacy of the Japanese people influences the use of healthcare measures for which the evidence is controversial and not well established Representative of such healthcare measures would be the area of healthcare grouped as complementary therapies [11,12]. Studies in the United States suggest that people with high health literacy tend to use complementary therapies at a higher rate [13,14]. In contrast, to our knowledge, no such studies have been reported from East Asian countries including Japan, China and South Korea.    One typical example of complementary therapies with insufficiently established evidence that has been used by some percentage of the Japanese population is acupuncture therapy. It has been reported that in Japan, approximately 5–7% of people receive acupuncture, at least once a year [15,16]. Musculoskeletal problems were the most common condition for which acupuncture was used (approximately 80%), and referral by family or friends was the most common reasons for people receiving acupuncture (approximately 60%) [16]. Around the 2000s, it was unknown to what extent information on the evidence for acupuncture was widespread within the Japanese population; additionally, it was unclear how much their health literacy played a role in their decision to use acupuncture for their intended condition. Incidentally, existing survey reports suggest that Japanese users of acupuncture tend to have lower education levels [15,16], but these results were probably confounded by age factor because the older population, who were relatively less educated, were more likely to use acupuncture than the younger [16]. Moreover, the relationship between health literacy and education levels has been controversial among scholars in Japan [9,10,17].  Recently, studies have found some evidence of the clinical effectiveness and safety of  acupuncture. Albeit insufficient, there are some positive conclusions regarding acupuncture for several health conditions like chronic low back pain and headaches in the Cochrane Database of Systematic Reviews [18–23]. Furthermore, several evidence-based clinical practice guidelines have been developed in Japan that include recommendations for acupuncture [24]. Under these circumstances, it remains unknown whether people with high health literacy adequately scrutinize the evidence, affirm acupuncture for the conditions for which evidence is shown, and consequently receive this treatment.  In this study, we conducted a web-based questionnaire survey in Japan to address the gap in this body of research. The aim of the study was to investigate the relationship between people’s health literacy and their health information sources and their attitudes toward acupuncture. In addition, this study aimed to understand the interrelationships among multiple factors such as sociodemographic characteristics, health literacy, health status, information sources, attitudes toward acupuncture and experience of acupuncture. Finally, to the WHO policy which encourages member states to provide individuals with reliable information on the benefits and risks of integrating traditional and complementary medicine into their healthcare system [25], our study can contribute insights for further understanding of and research on the relevant education measures for the public in terms of health literacy.  (Lines 45–106) |
| Within the Methodology: The study engaged 1600 participants. The participant selection procedure demands more excellent elucidation, accompanied by articulating any instances of participant attrition. It is imperative to consider the integration of additional statistical analyses, such as logistic regression. | Thank you for your comment. In the first round of peer review, following the suggestion of Reviewer #1, we have described the participant selection procedure in detail in "Study populations and procedures" in the Materials and Methods section. We would like to explain here the participant attrition.  As we stated in the first paragraph of the Results section, the total number of those who agreed to participate at the pre-survey stage was 3,292. As we also stated in the last paragraph of “Study populations and procedures” in the Materials and Methods section, the respective quota was closed automatically when 160 respondents in each age and sex group responded. Therefore, the 1,692 people who did not respond to the questionnaire include not only those who declined to respond but also those who were willing to respond but could not because the corresponding quota was already filled and closed. The breakdown of the reasons of the non-response of these 1,692 non-respondents was not disclosed by the internet research company Mellinks. We believe that this is an inherent limitation of panel-based studies.  Accordingly, we added a sentence explaining this insufficiency in the “Limitation of the study” section of the Discussion.  Incidentally, we would be grateful if you could also refer to our correspondence with Reviewer #3 in the second round of peer review, regarding the completion and response rates (see below):  *“This is a panel-based study, and even the 3,292 participants in the pre-survey stage were survey monitors who were pre-registered with the research firm. This has already been described in the sub-section, “Study populations and procedures” in Materials and methods.*  *Thus, the completion rate is quite different from the response rate in surveys such as random-digit dialing. When all quotas of age and sex were filled (in total 1,600 respondents), the survey automatically ended. Thereafter, the completion rate (CHERRIES checklist [22]) was calculated to be 48.6%. The process for completing this survey is also described in “Study populations and procedures” in the Materials and methods section.”*  As for statistical analysis in this study, we focused on correlations (or interrelationships) among many factors such as sociodemographic characteristics, health literacy, information sources, health status, attitudes toward acupuncture and experience of receiving acupuncture, rather than on predicting factors for one dichotomous event of positive/negative attitude toward acupuncture or experience of receiving acupuncture. This is the reason we did not show results of a logistic regression analysis. Path analysis has an advantage in showing complicated possible interactions among multiple observed variables, and it is widely used in studies in the behavioral sciences and psychology. We used path analysis and tried to illustrate direct and indirect relationships among multiple variables and identify the possible significant pathways related to health literacy and attitudes/behavior regarding acupuncture.  To make our intention clearer, we have modified the text in the Materials and Methods section.  If you believe that we still must consider additional statistical analyses such as a logistic regression model under the conditions described above, we would appreciate it if you could advise us again with specific reasons for doing so. | [“Limitation of the study” in *Discussion*]  This study had some limitations. First, because the number of respondents in each age and sex group was 160, it was not proportional to the population structure of Japan. Second, the respondents were not selected through random sampling, which is a common issue in web-based surveys using panel respondents. Third, it is difficult to know the classification and reasons of those who agreed to respond but were not included among the respondents (1,692 in this study), a limitation that is inherent to panel-based studies. Fourth, we did not collect data on the respondents’ income, which may have influenced the path analysis, particularly in terms of education, health literacy, and experience of acupuncture. Fifth, we did not focus on the relationship and influence of the type of comorbidities because the respondents were not proportional to the Japanese population, as mentioned above, and morbidity was less prevalent than we expected. Sixth, for the 25.4% of respondents who had received acupuncture in their lifetime, their attitudes toward this therapy might have been influenced by actual positive or negative experiences, rather than their health literacy. However, detailed questions on how those who received acupuncture in the past felt about the treatment were not included in this survey. Seventh, the measurement of health literacy was based on self-reporting, which may introduce biases, such as recall bias and over- or underestimation.  Despite these limitations, this is the first study to assess the relationship between health literacy and attitudes toward acupuncture, and it provides useful suggestions for the future of health education.  (Lines 476–495)  [“Data analysis” in *Materials and Methods*]  Additionally, we performed a path analysis to examine direct and indirect interrelationships among sociodemographic factors (sex, age, and educational attainment), health literacy, the number of information sources, health status, and experience and attitudes toward acupuncture among the participants.  (Lines 199–202) |
| Regarding the Discussion: The authors acknowledge that the study's participant cohort does not exhibit a representative cross-section of the broader Japanese populace. Consequently, it is recommended to revisit the formulation of the article title to reflect this limitation accurately. | Thank you for your comment. Considering that the cohort was not proportional to the population structure of Japan, we refrained from using phrase such as “Japanese people’s health literacy.” Additionally, in response to the suggestions provided by Reviewer #1, we have re-evaluated the title to make it more suitable for a research article. | [*Title*]  Relationship between health literacy and attitudes toward acupuncture: A web-based cross-sectional survey with a panel of Japanese residents  (Lines 1–2) |
| **Reviewer’s comments**  **(Reviewer #1)** | **Response to Reviewer** | **How we revised the manuscript** |
| It is a very minor issue; however, I would like to suggest changing the title to be in the form of a research-article.  Please review below:  https://blog.wordvice.com/how-to-write-the-perfect-title-for-your-research-paper/ | Thank you for your suggestion. Referring to the site you recommended, we modified the title to better reflect the study’s content and make it more suitable for a research article. | [*Title*]  Relationship between health literacy and attitudes toward acupuncture: A web-based cross-sectional survey with a panel of Japanese residents  (Lines 1-2) |
